# Short stature and vaginal dinoprostone as independent predictors of composite maternal-newborn adverse outcomes in induction of labor after one previous cesarean: a retrospective cohort study

**DOI:** 10.1186/s12884-024-06650-5

**Published:** 2024-07-01

**Authors:** Sze Ping Tan, Saniyati Badri Bashirudin, Rajeev Kumar Rajaratnam, Farah Gan

**Affiliations:** 1 Department of Obstetrics and Gynecology, Faculty of Medicine, University Malaya, Jalan Profesor Diraja Ungku Aziz, Kuala Lumpur, 50603 Malaysia; 2 Deparment of Obstetrics and Gynecology, North Middlesex University Hospital NHS Trust, Sterling Way, London, N18 1QX UK

**Keywords:** Labor induction, Previous cesarean, Predictor, Foley, Dinoprostone, Emergency cesarean

## Abstract

**Background:**

The rates of labor induction and cesarean delivery is rising worldwide. With the confluence of these trends, the labor induction rate in trials of labor after cesarean can be as high as 27-32.7%. Induction of labor after one previous cesarean (IOLAC) is a high-risk procedure mainly due to the higher risk of uterine rupture. Nevertheless, the American College of Obstetricians and Gynecologists considers IOLAC as an option in motivated and informed women in the appropriate care setting. We sought to identify predictors of a composite of maternal and newborn adverse outcomes following IOLAC.

**Methods:**

The electronic medical records of women who delivered between January 2018 to September 2022 in a Malaysian university hospital were screened to identify cases of IOLAC. A case is classified as a composite adverse outcome if at least one of these 11 adverse outcomes of delivery blood loss ≥ 1000 ml, uterine scar complications, cord prolapse or presentation, placenta abruption, maternal fever (≥ 38 ^0^C), chorioamnionitis, intensive care unit (ICU) admission, Apgar score < 7 at 5 min, umbilical artery cord artery blood pH < 7.1 or base excess ≤-12 mmol/l, and neonatal ICU admission was present. An unplanned cesarean delivery was not considered an adverse outcome as the practical management alternative for a clinically indicated IOLAC was a planned cesarean. Bivariate analysis of participants’ characteristics was performed to identify predictors of their association with composite adverse outcome. Characteristics with crude *p* < 0.10 on bivariate analysis were incorporated into a multivariable binary logistic regression analysis model.

**Results:**

Electronic medical records of 19,064 women were screened. 819 IOLAC cases and 98 cases with composite adverse outcomes were identified. Maternal height, ethnicity, previous vaginal delivery, indication of previous cesarean, indication for IOLAC, and method of IOLAC had *p* < 0.10 on bivariate analysis and were incorporated into a multivariable binary logistic regression analysis. After adjustment, only maternal height and IOLAC by vaginal dinoprostone compared to Foley balloon remained significant at *p* < 0.05. Post hoc adjusted analysis that included all unplanned cesarean as an added qualifier for composite adverse outcome showed higher body mass index, short stature (< 157 cm), not of Chinese ethnicity, no prior vaginal delivery, prior cesarean indicated by labor dystocia, and less favorable Bishop score (< 6) were independent predictors of the expanded composite adverse outcome.

**Conclusion:**

Shorter women and IOLAC by vaginal dinoprostone compared to Foley balloon were independently predictive of composite of adverse outcome.

## Synopsis

Shorter stature and dinoprostone labor induction are independent predictors of a composite maternal-newborn adverse outcome excluding unplanned cesarean delivery.

## Introduction

Data on the cesarean rate from 154 countries from 1990 to 2018 shows that the global cesarean delivery rate is rising in all regions, with the greatest increase of 44.9% in Eastern Asia [1]. National Health Service (NHS) England maternity statistics data shows induction of labor (IOL) rates have also increased, from 18.3% in 1989-90 to 34.4% by 2020 − 21 [2]. With the confluence of these trends, the IOL rate in trials of labor after cesarean (TOLAC) can be as high as 27-32.7% [3, 4]. 

Induction of labor after one previous cesarean (IOLAC) is a high-risk procedure mainly due to the higher risk of uterine rupture; the scar rupture rate is as high as 2.5% with the use of prostaglandins compared to a rate of 0.5% with spontaneous onset of labor and of 0.2% without a trial of labor [5]. Nevertheless, the American College of Obstetricians and Gynecologists (ACOG) considers IOLAC as an option in motivated and informed women in the appropriate care setting [6]. 

Findings of recent trials show that the unplanned cesarean rate can be as high as 59% [7] to 69% [8] after IOLAC. The adverse outcomes associated with TOLAC, and more so IOLAC, is an area of concern.

Several factors have been widely reported in the literature for having higher risk of morbidity following TOLAC. Among these are previous uterine rupture [9–14], myomectomy involving entry into the endometrial cavity [15, 16], inter-delivery interval < 16 months [17–20], grandmultiparity [21–27], labor induction especially with prostaglandins [5, 28–32], and labor dystocia [33–36]. Oxytocin use during TOLAC was not associated with worse maternal or neonatal outcomes in patients that had uterine rupture [37]. Risk calculators have been developed to predict uterine rupture in TOLAC however none are clinically reliable at present [38]. Studies specifically addressing IOLAC are sparse.

We aim to describe a contemporary cohort of women who underwent IOLAC and to identify independent risk factors for the occurrence of a composite of adverse maternal-newborn outcomes. Identifying these factors that exclude the unplanned but otherwise uncomplicated caesarean from consideration could assist in the counseling of women who are especially motivated to achieve VBAC. The results could also inform care providers on the selection of women for IOLAC and on the method to induce labor. The findings should enhance patient-provider shared decision making to undertake IOLAC.

## Materials and methods

This was a retrospective cohort study. All women who delivered at University Malaya Medical Centre (UMMC) from January 1, 2018 to September 30, 2022 had their electronic medical record (hospital chart) individually reviewed by investigator SBB to identify cases of IOLAC. IOLAC cases had their data retrieved and transferred onto a Case Report Form. Electronic medical records of IOLAC cases with incomplete information on the required study data are excluded. This study was approved by the Medical Research Ethics Committee of University Malaya Medical Centre (UMMC-MREC) on February 8, 2022 (reference number 202,215 − 10,901). Individual consent was not required by the review board.

UMMC is a tertiary, state-funded, full-services hospital, with care provided free-of-charge or heavily subsidised. Our center is located in urban Kuala Lumpur, Malaysia, a middle-income and multi-ethnic Asian country. We have a delivery rate of 4–5 thousand births a year with cesarean delivery rate of 35–40% and labor induction rate of 25–30%.

In our center, if the membrane is intact and the cervix unfavourable (Bishop score ≤5), cervical ripening is predominantly by the use of the Foley balloon that is left in place for up to 24 h from insertion. The vaginal 3 mg dinoprostone tablet is sometimes used depending on the provider; with a maximum daily dose of 6 mg (two doses, at least six hours apart). If a favorable cervix has not been achieved a third dose may be inserted the following day, after discussion with the patient. With spontaneous membrane rupture and an unfavourable cervix, titrated oxytocin infusion or vaginal dinoprostone tablet is used. The oxytocin infusion solution is prepared by diluting 10 units of oxytocin in 500 ml Hartmann’s solution (oxytocin concentration of 20 milliunits/ml) The infusion rate is started at 2 milliunits/hour and the rate doubled every half an hour until a contraction rate of 3 to 4 every 10 min is achieved, after which the infusion rate is maintained to sustain an optimal contraction rate of 3 to 4 moderate to strong contractions at each 10 min interval. Our maximum oxytocin infusion rate is 16 milliunits/hour in women with a previous cesarean delivery. In the event of uterine tachysystole, hypertonus or hyperstimulation syndrome with associated concerning fetal heart rate, the infusion rate will be reduced or even stopped. In our center, the concurrent use of Foley balloon, dinoprostone, or oxytocin for IOL is not standard care and rarely done. Oxytocin to initiate or augment contractions is typically only started after rupture of membranes.

The inclusion criteria were one previous cesarean section, underwent IOL, term (≥ 37 weeks), singleton, live, and cephalic fetus at induction, and maternal age ≥ 18 years. In our center, a repeat cesarean was recommended for women with two or more previous cesareans.

The retrieved data of IOLAC cases was transcribed onto a Case Report Form. The Case Report Form’s data selection were guided by known predictors of vaginal birth after cesarean (VBAC) [39] after a trial of labor (typically after spontaneous labor). Short maternal stature was defined as height of less than 157 cm, the median height for our population. The selected adverse maternal outcomes for the composite, were delivery blood loss ≥ 1000 ml [40], intensive care unit admission, uterine scar rupture and dehiscence, hysterectomy, umbilical cord prolapse, fever, chorioamnionitis, placental abruption and neonatal outcomes of admission to neonatal intensive care unit and the indication for admission, cord artery blood pH and base excess, and Apgar score at 5 min. These outcomes were systematically retrieved, verified and abstracted onto the Case Report Forms.

As planned cesarean delivery was the logical alternative to a medically indicated IOLAC [41], arguably a straightforward, albeit unplanned cesarean delivery without complication need not be considered as an adverse event. In this study, cases of unplanned cesarean delivery were excluded from the composite of adverse maternal–newborn outcomes if they did not also have at least one other adverse outcome already included in the composite.

Our target sample size was justified thus: trials have reported an unplanned cesarean rate of 50–60% after Foley balloon IOLAC [7, 8]. We presumed a smaller 12.5% composite adverse outcome rate that excluded uncomplicated cesareans. We anticipated 10 independent variables for the multivariable binary logistic regression analysis. To fulfil the 10 events per variable rule [42, 43], we would need at least 100 composite adverse outcome cases which could be expected to be found in 100/0.125 = 800 IOLAC cases.

Data were entered into SPSS (Version 26, IBM, SPSS Statistics). To identify independent predictors of the composite of adverse outcomes, bivariate analyses using the t-test was used to compare means of normally distributed continuous data, the Mann-Whitney U test for ordinal, or non-normally distributed data and Chi-square test for categorical data, dichotomized to composite adverse outcome present or absent. Variables with *p* < 0.10 on bivariate analysis were then included for multivariable binary logistic regression analysis to identify independent risk factors. For the adjusted analyses, 2-sided *p* < 0.05 was taken as a level of significance.

## Results

**Fig. 1:**
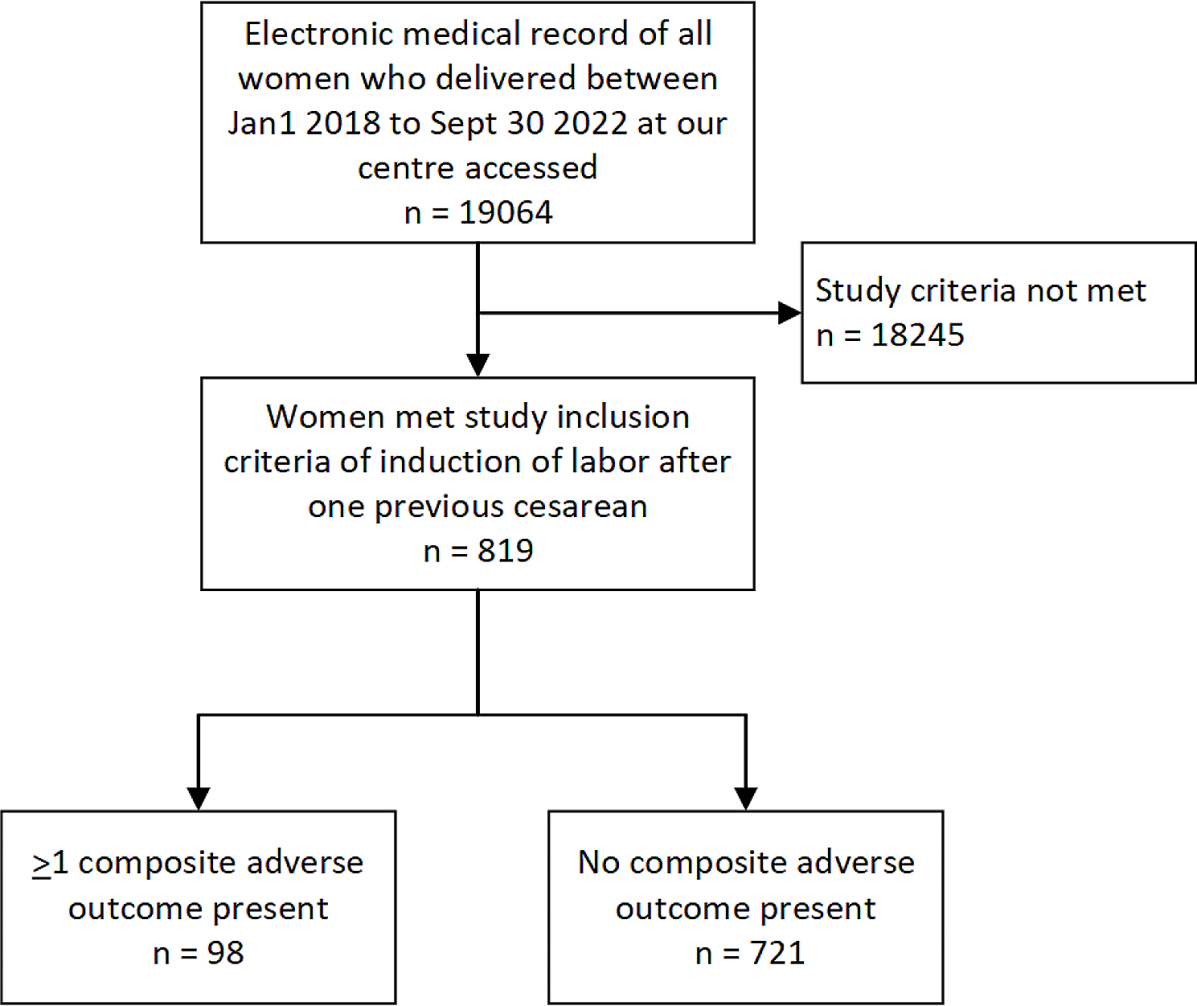
Flow chart for a retrospective study on independent predictors of composite adverse outcome (excluding unplanned cesarean) following induction of labor after one cesarean

Figure 1 depicts the flow through the study. From January 1, 2018 to September 30, 2022, 19,064 deliveries were recorded in our center. 819 women who had IOLAC were identified, of whom 98 had at least one adverse outcome in the composite list.

Table 1 shows the characteristics for the entire study population of 819 IOLAC cases. Basic demographics, selected obstetric history, and obstetric information on the index IOLAC pregnancy are shown.

**Table 1:** Characteristics of women who had induction of labor after one previous cesarean

Maternal Characteristics	*n* = 819
**Demographics**	
Maternal age (years)	32.4 ± 3.9
Body mass index (kg/m^2^)	30.9 ± 5.2
Height (cm)	157 [153–160]
Ethnicity	
Malay	593 (72.4%)
Chinese	73 (8.9%)
Indian & others	153 (18.7%)
Indian	94 (11.5%)
Others^a^	59 (7.2%)
**Obstetric history**	
Parity	
Parity 1	530 (64.7%)
Parity 2	164 (20.0%)
Parity ≥ 3	125 (15.3%)
Previous cesarean indication	
Failure to progress	253 (30.9%)
Non-reassuring fetal status^b^	341 (41.6%)
Others^c^	225 (27.5%)
Diabetes in pregnancy	366 (44.7%)
Hypertension in pregnancy	58 (7.1%)
Hemoglobin level pre-delivery (g/dl)	11.8 ± 1.1
Gestational age at induction (weeks)	38.7 ± 1.1
Bishop score at induction	6 [5–8]
Indication for induction	
Diabetes in pregnancy	256 (31.3%)
Non-reassuring fetal status^d^	214 (26.1%)
Prolonged pregnancy > 39 weeks	125 (15.3%)
Prelabour rupture of membrane	83 (10.1%)
Large for gestational age	70 (8.5%)
Others^e^	71 (8.7%)
**Labor information**	
Induction method	
Foley	591 (72.2%)
Prostaglandin	68 (8.3%)
Amniotomy and/or oxytocin	160 (19.5%)
Birth weight (kg)	3.058 ± 0.396

Data expressed as mean ± standard deviation, median [interquartile range] and number (%)

^a^ Includes Malaysian native tribes, Indonesian, Thai, Burmese, Bangladeshi, Sri Lankan, Yemeni, Sudanese and Nigerian

^b^ Includes abnormal fetal heart rate tracing, fetal growth restriction and abnormal dopplers

^c^ Includes non-cephalic presentation, hypertension in pregnancy, placenta previa, large for gestational age, maternal request, teenage pregnancy

^d^ Includes small for dates or growth restriction, oligohydramnios, abnormal Doppler studies, reduced fetal movement but fetal heart rate tracing must be reassuring at induction

^e^ Includes fetal anomaly, thrombocytopenia in pregnancy, gestational proteinuria, cholestasis at term

Table 2 illustrates the incidence of maternal-newborn outcomes. There were 463/819 (56.5%) unplanned cesarean after IOLAC, with 391/463 (84.4%) of them without any of the 11 selected adverse outcomes within the composite. Postpartum hemorrhage (≥ 1000 ml) [40] occurred in 39/819 (4.8%), cord accidents in 3/819 (0.37%), maternal admission to the intensive care unit in 4/819 (0.5%), uterine scar complications in 4/819 (0.5%) of which 2/819 (0.2%) were full thickness scar rupture, hysterectomy in 2/819 (0.24%), Apgar score at 5 min < 7 in 3/819 (0.4%), cord arterial blood pH < 7.1 in 20/819 (2.4%), base excess ≤ -12 in 16/819 (2.0%), and admission to the neonatal intensive care unit in 32/819 (3.9%), mostly for respiratory distress due to transient tachypnoea of the newborn (14/32, 43.8%), presumed sepsis (8/32, 25.0%), and congenital pneumonia (6/32, 18.8%). There was a solitary newborn who had hypoxic-ischemic encephalopathy.

**Table 2:** Adverse outcomes following induction of labor after one previous cesarean

Outcome	*n* = 819
**Maternal**	
Unplanned cesarean	463 (56.5%)
Without another adverse outcome	391 (47.7%)
With at least one other adverse outcome	71 (8.7%)
Postpartum hemorrhage	
≥ 1000 mL^1^	39 (4.8%)
Intensive care admission	4 (0.5%)
ICU admission indication	
Uterine incision extension at unplanned cesarean, uterine atony, hysterectomy, 4.5 L blood loss	1 (25%)
Cervical tears at spontaneous vaginal delivery, uterine atony, 4 L blood loss	1 (25%)
Severe birth canal injury with uterine artery transection at vacuum delivery, hysterectomy, 8 L blood loss	1 (25%)
Uterine atony at unplanned cesarean, B-Lynch suture, uterine arteries ligation, blood 2.5 L	1 (25%)
Hysterectomy	2 (0.2%)
Uterine scar complication	4 (0.5%)
Rupture	2 (0.2%)
Dehiscence	2 (0.2%)
Umbilical cord complications	3 (0.4%)
Prolapse	2 (0.2%)
Presentation	1 (0.1%)
Maternal fever ≥ 38 ^0^C	9 (1.1%)
Chorioamnionitis^2^	7 (0.9%)
Abruption^3^	2 (0.2%)
**Newborn**	
Apgar at 5 min < 7	3 (0.4%)
Cord artery blood	
pH < 7.1	20 (2.4%)
Base excess (mmol/l) ≤ − 12	16 (2.0%)
Neonatal intensive care unit (NICU) admission^4^	30 (3.7%)
NICU admission indication	
Respiratory distress	14 (46.6%)
Presumed sepsis	8 (26.7%)
Congenital pneumonia	6 (20.0%)
Hypoxic-ischemic encephalopathy	1 (3.3%)
Presumed birth asphyxia	1 (3.3%)

Data displayed as number (%)

^1^Blood loss estimation in our centre was made by visual assessment summing up blood loss in gauzes, swabs, pad, and drapes, and blood volume in the suction canister where applicable

^2^Chorioamnionitis is clinically diagnosed when maternal fever (≥ 38 ^0^C) is associated with two other maternal or fetal signs of systemic inflammation including maternal tachycardia, uterine tenderness, offensive amniotic fluid, leucocytosis, or fetal tachycardia

^3^Placental abruption is usually clinically diagnosed where there was a combination of unexplained vaginal bleeding, uterine pain and tenderness, fetal heart rate abnormality, placental separation and the presence of a retroplacental clot at delivery

^4^Two neonatal NICU admissions (one admission indicated by lupus and the other indicated by neonatal dysmorphic features) were not included as these indications were not plausibly relevant to labour induction

Table 3 lists the variables for bivariate analysis. Five of these variables, maternal height, previous vaginal delivery, indication of previous cesarean, indication of IOLAC and method of IOLAC emerged with bivariate analysis *p* < 0.1. After adjusted analysis, two independent predictors of composite adverse outcomes remained (significance level set at *p* < 0.05), namely height < 157 cm and IOLAC by vaginal dinoprostone compared to Foley balloon.

**Table 3:** Risk factors for composite adverse maternal-newborn outcomes following induction of labor after one previous cesarean (IOLAC) on bivariate and after multivariable binary logistic regression analysis

	Adverse Outcome	No Adverse Outcome	*p*-value	RR (95% CI)	AOR (95% CI)	*p*-value
	(*n* = 98)	(*n* = 719)				
Maternal demographics						
Maternal age (years)	32.6 ± 3.9	32.8 ± 4.0	0.743			
Body mass index (kg/m^2^)	30.8 [28.0–35.1]	30.5 [27.2–33.8]	0.147			
Obese (BMI ≥ 30)	56 (57.1%)	382 (53.0%)	0.438	1.08 (0.90–1.30)		
Height (cm)	154 [152–160]	157 [153–160]	0.017			
Height ≥ 157 cm^1^	39 (39.8%)	378 (52.4%)	0.019	0.76 (0.59–0.98)	0.58 (0.38–0.91)	0.017
Ethnicity			0.018			0.454
Malay	70 (71.4%)	523 (72.5%)			1.86 (0.57–5.25)	0.231
Indian and Other	23 (23.5%)	130 (18.0%)			1.53 (0.58–4.02)	0.387
Indian	9 (8.8%)	85 (11.9%)				
Other	14 (13.7%)	45 (6.3%)				
Chinese	5 (5.1%)	68 (9.4%)			^2^	
Obstetric history						
Parity			0.207			
Parity 1	71 (72.4%)	459 (63.7%)				
Parity 2	14 (14.3%)	150 (20.8%)				
Parity ≥ 3	13 (13.3%)	112 (15.5%)				
Previous vaginal delivery	27 (27.8%)	262 (36.3%)	0.088	0.76 (0.54–1.06)	0.69 (0.42–1.15)	0.154
Indication of previous cesarean			0.074			0.200
Failure to progress	40 (40.8%)	213 (29.5%)			^2^	
Non-reassuring fetal status	35 (36.7%)	305 (42.3%)			0.69 (0.42–1.14)	0.144
Other	22 (22.4%)	203 (28.2%)			0.63 (0.35–1.12)	0.115
At index pregnancy^3^						
Diabetes in pregnancy	43 (43.9%)	323 (44.6%)	0.863	0.98 (0.77–1.24)		
Hypertension in pregnancy	6 (6.1%)	52 (7.2%)	0.693	0.85 (0.38–1.92)		
Hemoglobin at IOLAC^4^ (g/dl)	11.8 [10.8–12.6]	11.8 [11.1–12.6]	0.164			
Gestation at IOLAC^4^ (weeks)	38.7 [38.0–39.9]	38.6 [37.9–39.7]	0.174			
IOL indication			0.040			0.090
Diabetes in pregnancy	30 (30.6%)	226 (31.3%)			^2^	
Non-reassuring fetal status	17 (17.3%)	197 (27.3%)			0.69 (0.37–1.31)	0.259
Prolonged pregnancy	20 (20.4%)	105 (14.6%)			1.55 (0.83–2.90)	0.170
PROM^5^	8 (8.2%)	75 (10.4%)			0.86 (0.33–2.20)	0.748
Suspected LGA^6^	15 (15.3%)	55 (7.6%)			1.98 (0.98–3.99)	0.058
Others	8 (8.2%)	63 (8.7%)			1.11 (0.47–2.60)	0.812
Bishop score at induction	6 [5–8]	6 [5–8]	0.938			
Bishop score ≥ 6	61 (62.2%)	475 (65.9%)	0.478	0.95 (0.80–1.11)		
Induction method			0.053			0.030
Foley	69 (70.4%)	522 (72.4%)			^2^	
Dinoprostone	14 (14.3%)	54 (7.5%)			2.44 (1.23–4.84)	0.011
Oxytocin or amniotomy	15 (15.3%)	145 (20.1%)			0.92 (0.46–1.82)	0.806

Data expressed as mean ± standard deviation, median [interquartile range] or number (%). Student t-test was used for analysis of continuous normally distributed data, Mann Whitney U test used for ordinal and non-parametric data and Chi Square test used for categorical or nominal data. Multivariable binary logistic regression was performed incorporating variables with *p* < 0.1 on bivariate analyses to identify independent predictors of composite adverse outcome (any one of delivery blood loss ≥ 1000 mL, intensive care unit admission, uterine scar rupture and dehiscence, hysterectomy, umbilical cord prolapse, fever (≥ 38 ^0^C), chorioamnionitis, admission to neonatal intensive care unit, cord artery blood pH (< 7.1) and base excess (< -12 mmol/l), and Apgar score at 1 (< 4) and 5 (< 7) minutes)

^1^Median cut off for the study population

^2^Referent group

^3^Pregnancy of induction of labor after cesarean

^4^Induction of labor after one previous cesarean

^5^Prelabor rupture of membranes

^6^Large for gestational age: Clinical diagnoses made by their care providers and verified as at least probable at the individual data retrieval (estimated fetal weight is above the 90th centile for gestational age, fetal abdominal circumference ≥ 350 mm and/or estimated fetal weight ≥ 3500 g by ultrasound, typically within the last week of decision to induce)

### Post hoc analysis

Post hoc, we sought to evaluate independent predictors for composite adverse outcome after IOLAC that included unplanned cesareans as a component of the composite. With this analysis there were 489 cases positive for composite adverse outcomes. Of these, 463/489 (94.7%) had unplanned cesarean and only 72/463 (15.5%) were unplanned cesarean with at least one of the 11 other components of the composite. Following vaginal delivery after IOLAC, there were 26/356 (7.3%) cases positive for composite adverse outcomes.

Table 4 showed the bivariate and multivariable binary logistic regression analyses (variables with *p* < 0.1 incorporated into the model) for the expanded composite adverse outcomes that included all unplanned cesarean. Nine variables had *p* < 0.1 after bivariate analysis. Following adjustment, six variables (higher body mass index, short stature (< 157 cm), not of Chinese ethnicity, no prior vaginal delivery, prior cesarean indicated by labor dystocia and less favorable Bishop score (< 6) were independent predictors of the expanded composite adverse outcome. Maternal age, gestational age at IOLAC, and method of IOLAC were not significant (set at *p* < 0.05) after adjustment.

**Table 4:** Composite adverse outcomes including of unplanned cesarean

	Composite adverse outcomes	No composite adverse outcomes	*p*-value	RR (95% CI)	AOR (95% CI) for *p* < 0.05	*p*-value
	(*n* = 489)	(*n* = 330)				
**Maternal demographics**						
Maternal age (years)	32.31 ± 3.7	33.39 ± 4.2	< 0.001		1.03 (0.98–1.07)	0.303
Body mass index (kg/m^2^)	31.1 [27.9–34.2]	29.4 [26.9–32.6]	< 0.001		1.03 (1.02–1.08)	0.003
BMI ≥ 30	286 (58.5%)	152 (46.1%)	< 0.001	1.23 (1.09–1.34)		
Height (cm)	156 [153–160]	157 [153–161]	0.010			
Height ≥ 157 cm^1^	232 (47.4%)	185 (56.1%)	0.016	1.15 (1.03–1.29)	0.70 (0.51–0.97)	0.030
Ethnicity			< 0.001			< 0.001
Malay	339 (69.3%)	254 (77.0%)			2.49 (1.42–4.367)	0.001
Indian and Other	121 (24.7%)	32 (9.7%)			4.71 (2.45–9.08)	< 0.001
Indian	75 (15.3%)	19 (5.8%)				
Other	46 (9.4%)	13 (3.9%)				
Chinese	29 (5.9%)	44 (13.3%)			^2^	
**Obstetric history**						
Parity			< 0.001			< 0.001
Parity 1 (one previous cesarean only)	384 (78.5%)	146 (44.2%)			^2^	
Parity 2	71 (14.5%)	93 (28.2%)			0.30 (0.20–0.45)	< 0.001
Parity ≥ 3	34 (7.0%)	91 (27.6%)			0.14 (0.08–0.24)	< 0.001
Previous cesarean indication			< 0.001			0.012
Failure to progress	181 (37.0%)	72 (21.8%)			^2^	
Non-reassuring fetal status	198 (40.5%)	143 (43.3%)			0.67 (0.45–0.92)	0.040
Other	110 (22.5%)	115 (34.8%)			0.54 (0.35– 0.82)	0.004
**At index pregnancy**						
Diabetes in pregnancy	223 (45.6%)	143 (43.3%)	0.522	1.04 (0.92–1.16)		
Hypertension in pregnancy	36 (7.4%)	22 (6.7%)	0.704	1.04 (0.85–1.29)		
Hemoglobin at IOLAC^3^ (g/dl)	11.9 [11.1–12.6]	11.8 [11.1–12.6]	0.649			
Gestation at IOLAC^3^ (weeks)	38.7 [37.9–39.9]	38.4 [37.9–39.6]	0.051		1.03 (0.89–1.18)	0.738
IOL^4^ indication			0.117			
Non-reassuring fetal status	111 (22.7%)	103 (31.2%)				
Diabetes in pregnancy	162 (33.1%)	94 (28.5%)				
Prolonged pregnancy	80 (16.4%)	45(13.6%)				
PROM^5^	47 (9.6%)	36 (10.9%)				
Suspected LGA^6^	44 (9%)	26 (7.9%)				
Others	45 (9.2%)	26 (7.9%)				
Bishop score at induction	6 [5–8]	6 [5–8]	< 0.001			
Bishop score ≥ 6	290 (59.3%)	246 (74.5%)	< 0.001	0.77 (0.69– 0.86)	0.58 (0.40–0.83)	0.003
Induction method			0.014			0.503
Foley	371 (75.9%)	220 (66.7%)			^2^	
Prostaglandin	37 (7.6%)	31 (9.4%)			0.82 (0.45– 1.48)	0.503
Oxytocin or amniotomy	81 (16.6%)	79 (23.9%)			0.85 (0.56–1.28)	0.436

Data are expressed as mean ± standard deviation, median [interquartile range] or number (%). Student t-test was used for analysis of continuous normally distributed data, Mann Whitney U test used for ordinal and non-parametric data, and Chi Square test used for categorical or nominal data. Multivariable binary logistic regression was performed incorporating variables with *p* < 0.01 on bivariate analyses to identify independent predictors of composite adverse pregnancy outcome (at least one of unplanned cesarean, delivery blood loss ≥ 1000 mL, intensive care unit admission, uterine scar rupture and dehiscence, hysterectomy, umbilical cord prolapse, fever (≥ 38 ^0^C), chorioamnionitis, admission to neonatal intensive care unit, cord artery blood pH (< 7.1) and base excess (< -12 mmol/l), and Apgar score at 1 (< 4) and 5 (< 7) minutes)

^1^Median cut off for the study population

^2^Referent group

^3^Induction of labor after one previous cesarean

^4^Inductin of labor

^5^Prelabor rupture of membranes

^6^Large for gestational age

In a previous analysis from the same study population, we have found that obesity, short stature, no prior vaginal delivery, previous cesarean indicated by failure to progress, unfavorable Bishop score and ethnicity were independent predictors for unplanned cesarean after IOLAC [44]. These post hoc findings (Table 4) were reflective of the numerical dominance of the unplanned cesarean subpopulation, overwhelming the 11 other adverse events in the composite.

## Discussion

In our analysis on composite adverse maternal-newborn outcomes after IOLAC but specifically excluding uncomplicated cesarean deliveries, after multivariable binary logistic regression analysis, we identified two independent predictors for the composite adverse outcome; short maternal stature and labor induction using vaginal dinoprostone.

Short maternal stature (< 157 cm) was independently predictive of composite adverse outcome similarly to it being a risk factor for unplanned cesarean delivery after IOLAC [44, 45]. Our result corroborated the finding from a Swedish cohort study which reported maternal height of < 160 cm to be a risk factor of uterine rupture during TOLAC with OR 1.69 compared to patients > 160 cm tall [46]. In our study, short stature remained an independent predictor of the expanded composite adverse outcome that included all unplanned cesarean section. Machine learning models have also shown maternal height to significantly contribute to the prediction of successful VBAC [47]. 

Dinoprostone, compared to Foley induction, was also found to be predictive of the composite adverse outcomes after adjustment. Available research on IOLAC primarily centers on successful vaginal birth or risk of uterine rupture. Meta-analyses [39, 48] from sparse data on IOLAC methods did not reveal a superior induction method. A recent individual participant data meta-analysis of randomized controlled trials however found balloon catheters for cervical ripening in labor induction led to fewer adverse perinatal events compared to prostaglandins, although no exclusion was made based on previous cesarean delivery status [48]. Our findings contribute to the limited data on risk factors specific to complications after IOLAC.

In our bivariate analysis, previous vaginal delivery, indication of previous cesarean and indication of IOLAC emerged as potential predictors of adverse outcomes, but these were not significant after adjustment. These variables were similarly not significant in TOLAC studies assessing morbidity, except for large-for-gestational-age fetuses having shown an association with uterine rupture following cesarean delivery [17, 27]. 

Previous analysis from the same study population showed previous cesarean indicated by failure to progress and no prior vaginal delivery to be independent predictors of unplanned cesarean after IOLAC [44]. Unplanned cesarean without complication was excluded as a component of the composite of adverse outcomes in our primary analysis. This exclusion could be controversial as adverse psychosocial outcomes, including post-traumatic stress, health-related quality of life, experiences, infant-feeding, satisfaction, and self-esteem were negatively impacted by emergency cesarean section [49]. Even in well-motivated women with extensive counseling on the risk of failed IOLAC and unplanned cesarean, a degree of disappointment was likely when unplanned cesarean occurred [50]. However, our novel approach of excluding unplanned cesarean without complication from the composite adverse outcomes classification as the practical alternative to IOLAC is a planned cesarean, would be of value for care providers and women open to a different approach when looking at information to help decide on IOLAC.

### Research implication

Our findings of independent predictors of a composite adverse maternal-newborn outcomes add to the limited body of evidence on risk factors related to the performance and safety of IOLAC. Further very large scale confirmatory retrospective studies should increase the confidence on our findings and plausibly identify other risk factors missed as a result of Type 2 error. Large scale prospective studies with well-defined and consistently applied terms, focused on IOLAC subjects, will provide the highest quality data to identify independent predictors and allow for the development of robust calculators to give more precise estimates of the risk of adverse outcome to aid decision-making on IOLAC.

### Strengths and limitations

As to strength, we had a relatively large contemporary set of 819 IOLAC cases, with data individually abstracted directly from their medical records and a sample sufficiently large for robust multivariable binary logistic regression analysis based on the 10-event per variable rule [43]. Our independent predictors of composite adverse outcome after IOLAC were likely to be robust as they concurred with extensive meta-analysis findings from TOLAC studies [39] and from sparser data on unplanned cesarean births after IOLAC [44, 45]. Our IOLAC cases were identified and their data abstracted by a single clinician-investigator (SBB) who reviewed all the birth records.

We were limited by the number of composite adverse outcome at only 98 cases, which could have resulted in Type 2 error due to underpowering. The use of dinoprostone in cases plausibly at lower-risk in our practice may lead to the underestimation of its true impact on adverse outcomes despite adjustment to reduce confounding. Prostaglandin as a method of IOLAC may be regarded as controversial in the absence of conclusive safety results [5] but meta-analyses [39, 48] on IOLAC methods did not reveal a superior IOLAC method although the available data is sparse. We also did not retrieve the number of prostaglandins used in cases of adverse outcomes. Obstetric sphincter injury (OASIS) was not explored in our study; previous cesarean section increases the risk of OASIS [51] but OASIS does not appear to be associated with IOL per se [52]. We used delivery blood loss *≥* 1000 ml as an adverse maternal outcome for the composite instead of the need for blood transfusion, which could be a more objective and clinically useful measure. With a retrospective chart review, even from electronic medical records, the data could still be inaccurately or incompletely documented.

## Conclusion

Short maternal stature and vaginal dinoprostone tablet compared to Foley balloon induction are independent predictors of a composite of adverse maternal-newborn outcomes after IOLAC. These predictors could aid care providers and women in their shared decision making on IOLAC and on the method of induction, beyond the consideration of an unplanned cesarean as adverse outcome.

## Data Availability

The datasets used and analyzed during the current study are available from the corresponding author on reasonable request.
